# Nationwide Desert Highway Assessment: A Case Study in China

**DOI:** 10.3390/ijerph8072734

**Published:** 2011-06-30

**Authors:** Xuesong Mao, Fuchun Wang, Binggang Wang

**Affiliations:** Key Laboratory for Special Area Highway Engineering of Ministry of Education, Chang’an University, Xi’an 710064, China; E-Mails: wangdafc@126.com (F.W.); wangbinggang999@163.com (B.W.)

**Keywords:** highway engineering, desert assessment, index system, environmental protection

## Abstract

The natural environment affects the construction of desert highways. Conversely, highway construction affects the natural environment and puts the ecological environment at a disadvantage. To satisfy the variety and hierarchy of desert highway construction and discover the spatio-temporal distribution of the natural environment and its effect on highway construction engineering, an assessment of the natural regional divisions of desert highways in China is carried out for the first time. Based on the general principles and method for the natural region division, the principles, method and index system for desert highway assessment is put forward by combining the desert highway construction features and the azonal differentiation law. The index system combines the dominant indicator and four auxiliary indicators. The dominant indicator is defined by the desert’s comprehensive state index and the auxiliary indicators include the sand dune height, the blown sand strength, the vegetation coverage ratio and the annual average temperature difference. First the region is divided according to the dominant indicator. Then the region boundaries are amended according to the four auxiliary indicators. Finally the natural region division map for desert highway assessment is presented. The Chinese desert highways can be divided into three sections: the east medium effect region, the middle medium-severe effect region, and the west slight-medium effect region. The natural region division map effectively paves the way for the route planning, design, construction, maintenance and ongoing management of desert highways, and further helps environmental protection.

## 1. Introduction

**Figure 1:**
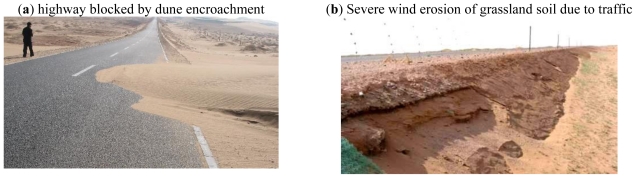
Interactive effects between highway engineering and the natural environment.

There are about 7,900 kilometers of desert highways in China, mainly distributed in the Xinjiang Ugur Autonomous Region, Ningxia Hui Autonomous Region, Neimengu Autonomous Region and Gansu Province and some other areas. The differences between desert highways and general highways are due to the effects of the arid climate, the wind sand and the temperature on highway construction engineering. Another difference is the shortage of suitable filling materials such as soils and stones for highway construction in desert regions, which makes it more difficult to construct a desert highway. A highway exists in the natural environment as a structure. The elements of the natural environment affect the highway construction engineering. At another hand, the construction affects the natural environment and puts the ecological environment at a disadvantage. Especially in desert regions the highways can always be blocked by the encroachment of sand dunes [Figure 1(a)], and the highway traffic can cause severe wind erosion of grassland soils [Figure 1(b)]. To satisfy the variety and hierarchy of the highway construction and discover the spatio-temporal distribution of the desert natural environment and its effects on highway construction, a desert highway assessment should be carried out, which will effectively pave the way for route planning, design, construction, maintenance and subsequent management of desert highways, and help formulate standards.

Desert highway assessment studies have been going on for many years. The earliest desert assessment can be dated back to Lamprey’s research in central western Sudan, when he introduced the desert encroachment theory [1], which laid the groundwork for providing a process for measuring the desertification problem. A provisional methodology for assessment and mapping of desertification was formulated [2,3] and is now used for local and regional assessment and mapping [4,5]. Most of the studies assess the desert based on the desert topography, vegetation coverage and wind erosion. The desert is divided into two kinds (fixed and mobile) or three kinds (fixed, part fixed or mobile), according to the extent of the activity of the sand dunes. China began a nationwide desertification assessment in the mid-1980s. Historically, China had focused on sandy desertification, which is caused by wind erosion [6]. This classification method was based on geo-morphological changes caused by blowing sand (such as sand dunes and sand mound formations, sand cover, and surface coarseness), the percentage of change in relation to the total area, and the annual rate of increase of desertified land. These classifications had been broadly used in sandy desertification assessment [7]. Based on hundreds of expert questionnaires, sandy desertification intensities were classified as slight, medium, severe, or extremely severe [8]. The main assessment indicators were bare sand ratio, vegetation coverage ratio, and soil texture, and the importance of each indicator was calculated by the Delphi weighted method.

However a desert highway assessment that comprehensively describes the natural highway distribution or single local highway distribution has not been reported until now at home or abroad. With the increase in highway construction, especially as a result of the Western Development Plan of China, highway construction in the desert regions had accelerated considerably and the highway mileage has increased yearly. Li Bin had put forward the idea of desert highway assessment in China. He designed the indicators as the moisture coefficient and aimed to determine the differences of the highway subgrade protective level in desert regions [9]. However, he only put forward the theory and the natural region division map for desert highway assessment was not drawn out. His studies were rough and could not be applied to actual highway construction. This paper borrows the idea of desert assessment method. Based on the general principle and method for the regional division, the principles, method and index system for the desert highway assessment are put forward by combining the desert highway construction and the azonal differentiation law. The method of combining both one dominant indicator and four auxiliary indicators is applied to divide the desert highway region distribution in China for the first time.

## 2. Principle and Method for Desert Highway Assessment

### 2.1. Principles of Desert Highway Assessment

1. Service principle: Desert highway assessment will serve the highway engineer in China. The objective of the assessment is clear, namely to provide the basic science data for balancing the highway construction and the protection of the natural environmental, to further promote the sustainable development of desert regions [10].
2. Practicability principle: The desert highway assessment process can comprehensively reflect the natural characteristics. It studies the effect of natural environment factors on the highway construction from the viewpoint of a real highway engineer. At the same time it synthesizes the differences between desert highways and general highways with respect to the route design, subgrade, pavement, and protection works.
3. Dialectical principle: All the factors, such as the sand physiognomy, sand strength, temperature annual difference, and the vegetation will affect the desert highway construction. They are related and correlative. The desert highway assessment divides the nationwide desert highway regions in China according to their similarity and the differences between natural factors. The regional divisions should not conflict with each other.
4. Absolute consistency: Every natural unit forms along history little by little. The desert highway assessment should discuss the reason and process of the desert region and pave the way for regional division. Only under the absolute consistency principle, can the correlative consistency of the divided or united desert unit be guaranteed during the highway region division.
5. Relative consistency: The relative consistency requires that the internal consistency should be maintained for the desert highway division. Different region unit levels are different in their consistency.
6. Combination of comprehensive and dominant factors: Any desert region is combined with several natural geological factors. The desert highway assessment should pay attention to analyze their modes of interaction, to process these factors and to recognize the real discipline of the regional differences. At the same time, the dominant factor should be distinguished among all other factors. This principle is balanced between the comprehensive and dominant factor.
7. Similar characteristics of highway engineering in desert regions: The principle of similar characteristics of highway engineering in desert regions is basic to define the indicators and level of highway desert assessment. The so-called highway engineering characteristics in desert regions are the general and global characteristics of all highway engineering under the effects of natural geological factors.

### 2.2. Methods for Desert Highway Assessment

1. The balance between the comprehensive principle and the dominant factor principle: The comprehensive principle requires us to analyze and to combine all the factors for defining the assessment index, which affect the highway engineering in desert regions. Further the important factors are selected such as the sand dune height, the blown sand strength, the vegetation coverage ratio and the annual average temperature difference, which are keys for the construction, maintenance, and running management of highway engineering [11,12].
2. The balance between the dominant indicator and the auxiliary indicators: For the desert highway assessment, there are several key factors to highway construction. It is impossible to apply all the factors for the highway desert division, which is also unnecessary. The dominant indicator uses the compromise mathematical function to express both the comprehensive character and the dominant character. The comprehensive state index of the desert highway is defined as the dominant indicator for region division. At the same time four auxiliary indicators are adopted to revise the division boundaries, which are defined as the sand dune height, the blown sand strength, the vegetation coverage ratio and the annual average temperature difference [13].

## 3. Index System of Highway Desert Assessment in China

The dominant indicator for assessing the desert comprehensive state must be based on a sufficient investigation and wide collection of field data. Then the advanced technology of GIS and related databases is used [14]. The function equation is established according to the real experience and data files, such as highway engineering standards and protocols. The equation is also combined with the real situation of the highway construction to select the important environmental factors, which are crucial to the highway construction in the desert regions. After the equation is established, every parameter is defined and calculated. The scale value and weighting coefficients are made clear based on the commensurability theory. Finally the comprehensive state index of every division region is obtained with the dominant and auxiliary indicators, which forms the natural region division map. Based on the order of importance, the natural region division map of the desert comprehensive state index is drawn. According to one dominant indicator (the desert comprehensive state index) and four auxiliary indicators (the sand dune height, the blown sand strength, the vegetation coverage ratio and the annual average temperature difference), the highway desert assessments are carried out.

### 3.1. The Definition of Important Indicators

The desert comprehensive state index means the effects of the natural environment factors on the highway construction in desert region. According to years of field experience, the natural environment factors are crucial to the route design, construction, maintenance and running management of highways, and mainly include the sand dune height, the blown sand strength, the vegetation coverage ratio and the annual average temperature difference.

1. The sand dune height: The sand dune height is one of the most significant indicators for analyzing the physiognomy features of blown sand regions. It can reflect the time of formation of the desert physiognomy and the scale of the sand dune growth. Generally the longer the time of formation of the sand dune is, the more height the sand dune has, due to the sand movement and sand accumulation under wind for a long time. On the contrary, the newly grown sand dunes are short with less sand accumulation. The sand dune height can also reflect the active strength of sand dune movement. Under the same wind flow and natural situation, the height difference is dominant in the active strength of the sand dune movement.
  The sand dune height affects the route design, construction and the later management of the highway engineering operations. The sand dune height directly affects the route layout of the highway and at the same time causes blown sand damage to the highway. During the later running management and maintenance, it is more difficult to maintain the highway in the desert environment, so the effect of the sand dune height on the highway is crucial to recommend the route layout plan and protect the highway from the desert damage.
  The height of sand dunes is expressed by the average value of all sand dune heights in a region. According to the 1:25,000,000 digital elevation model in China, the sand dune height distribution is not uniform, however most sand dune heights are within a fixed range, so the average height of sand dunes in a region can be adopted to represent the wave distribution of sand dunes.
2. The blown sand strength: The blown sand strength is defined as the cumulative velocity record of the blown sand during a rated time (month, season or year). It depends on the observation times and the observed value of the wind velocity every time, so its unit is m/(s·time unit). For the desert highway assessment in China, the time unit is 30 years and its unit is m/(s·year). The blown sand strength is one of the most important indicators for blown sand movement and is proportional to the wind velocity. It is crucial to the design of the desert highway and also to the subsequent management and maintenance. It will affect the route layout of the highway and causes desert damage. During the construction of highways in desert regions, more attention should be paid to the blown sand strength. It is defined by the sand diameter, the ground characteristics and the water content of the sand. The blown sand strength can indirectly reflect the wind velocity. This is the first time the blown sand strength is adopted as one of the indicators for desert highway assessment, which can direct the route layout of the highway and its latter running management and maintenance.
3. The vegetation coverage ratio: Vegetation is a general term for the plant life of a region. The vegetation coverage ratio is defined as the percent of the planimetric area of the vegetation (leaves, branches and stems) on the statistical region area. In desert regions, vegetation is rare, so the ratio is low and in some desert regions there is even no vegetation. However, the vegetation coverage can reflect the stability of a sand dune. Furthermore it affects the design, construction and protection of the highway, so it is crucial to evaluate the effect of the vegetation on the highway construction, recommend the optimal route layout and build different level protective measures into the highway engineering plans.
4. The annual average temperature difference: The annual average temperature difference means the difference between the maximum and the minimum of the average annual temperature in a region. It reflects the climate effect on the highway construction. The highway will have a route layout exposed to the natural climate, which includes the temperature and rainfall. In a general region, with the periodical changes of the temperature, the moisture in the subgrade will undergo frost and thaw cycles, so there can be frost boiling damage, but in the desert regions the rainfall is rare, so the effect of the rainfall on the subgrade is small and the temperature changes affect the pavement more. The asphalt pavement can suffer fatigue cracking under repeated exposure to high and low temperature cycles. At the same time, the high temperatures will lead to the asphalt ageing. The characteristics of the asphalt and its mixtures are different under high or low temperature. The requirements for asphalt in desert regions are high because the differences between the maximum and minimum temperatures are large. The annual average temperature difference in desert region directly affects the structure of the highway and its pavement material selection.

### 3.2. Mathematical Equation

The desert comprehensive state can reflect the effects of the natural factors on highway construction. It consists of the four aforementioned natural factors, such as the sand dune height, the blown sand strength, the vegetation coverage ratio and the annual average temperature difference. The four natural factors have different characteristics, content and dimensions, so the first step for establishing the function of desert comprehensive state index should be to normalize all the natural factors according to the comprehensive evaluation theory. Then the weight coefficient of every factor should be defined. The established equation is shown in Equation 1:

(1) $${NEP}_{i}=K_{h}\cdot M_{ih}+K_{p}\cdot M_{i\;p}+K_{f}\cdot M_{i\;f}+K_{t}\cdot M_{i\;t}$$

where: *NEP_i_* the state for the desired calculation unit;

*K_h_* the weight coefficient of the sand dune height;

*K_p_* the weight coefficient of the vegetation coverage ratio;

*K_f_* the weight coefficient of the blown sand strength;

*K_t_* the weight coefficient of the annual average temperature difference;

*M_ih_* the normal contribution of the sand dune height in the desired calculation unit;

*M_ip_* the normal contribution of the vegetation coverage ratio in the desired calculation unit;

*M_if_* the normal contribution of the blown sand strength in the desired calculation unit;

*M_it_* the normal contribution of the annual average temperature difference in the desired calculation unit.

The first step is to normalize the four natural factors, then calculate the normal contribution of every factor. Following the 1994 United Nations Convention to Combat Desertification (UNCCD), the Chinese Committee for Implementing UN Convention to Combat Desertification (CCICCD) organized a group of scientists to assess nationwide desertification [15,16]. The group studied wind erosion, water erosion, temperature and vegetation in addition to degradation by other driving factors. This study determined four desertification intensities (slight, medium, severe and extremely severe) and the scientists formulated a detailed assessment criteria for classification [16]. Considering the significance of every parameter, the scientists’ assessment criteria were based on past research results and extensive expert discussions (Table 1).

Then the weighting coefficient of every factor is defined as shown in Table 2. The values of every coefficient in Table 2 are defined by the scores of ten specialists, who are recognized in the highway design and construction area.

### 3.3. Calculation Process of the Four Auxiliary Indicators

**Figure 2:**
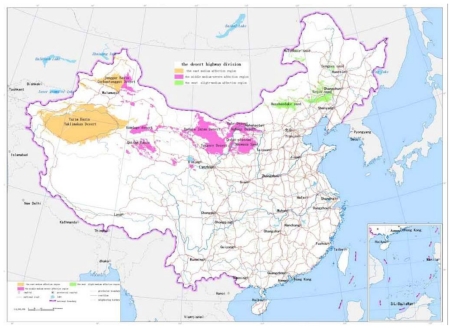
The map of the sand dune height.

**Figure 3:**
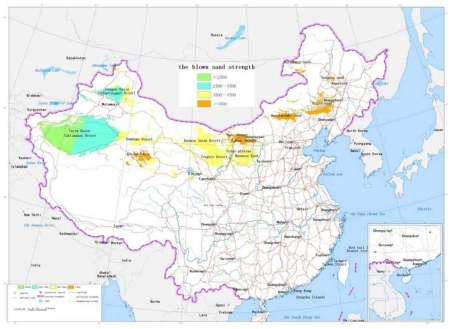
The map of the blown sand strength.

**Figure 4:**
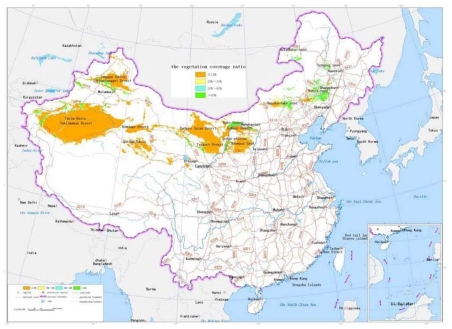
The map of the vegetation coverage ratio.

**Figure 5:**
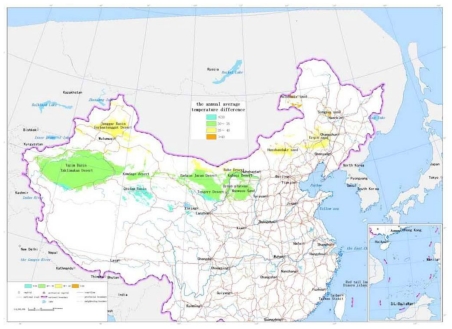
The map of the annual average temperature difference.

1. The sand dune height: The dedicated analysis tool for the sand dune height is developed based on the ArcInfo9.1 software and GIS module. The unit is selected as 5 kilometers square. Digital Elevation Model of 1:25 desert region in China are traversed one unit by one unit. The maximum value and average difference of elevation of every unit are calculated, which forms the distribution data of the sand dune height in China. According to Table 1, the scale value is calculated and the map of the sand dune height is drawn, as shown in Figure 2.
2. The blown sand strength: The weather data from the local weather stations in the desert regions are collected. The cumulative annual average strength of blown sand was calculated for a 30 year period. With the software ArcInfo9.1 and its “Geostatistical Analysis” module, the spatial distribution of blown sand strength is analyzed by the Cregeen interpolation method [17], which borrows from the spatial variation theory. Further the isopleth map of the blown sand strength is drawn. The average strength of blown sand in every region unit can then be calculated. According to the calculated results and the criteria of Table 1, the level value of every region unit is obtained and the map of the blown sand strength is drawn, as shown in Figure 3.
3. The vegetation coverage ratio: According to the 1:1,000,000 map of vegetation coverage in China, the percentage of vegetation coverage in every region unit can be calculated using the ArcInfo9 software. According to the calculated results and the criteria of Table 1, the level value of every region unit is obtained and the map of the vegetation coverage is formed, as shown in Figure 4.
4. The annual average temperature difference: The weather data are collected from the local weather stations in the desert regions. The annual average temperature difference is also calculated for 30 years. The maximum and minimum annual average temperatures in every region unit are obtained. With the software ArcInfo9.1 and its “Geostatistical Analysis” module, the spatial distribution of the annual average temperature in every region unit is analyzed. Further the isopleth map of the annual average temperature difference is drawn. The temperature difference in every region unit can be calculated. According to the calculated results and the criteria of Table 1, the level value of every region unit is obtained and the map of the annual average temperature difference is formed, as shown in Figure 5.

## 4. Natural Region Division Map for Desert Highway Assessment in China

By Equation 1, the Tables 1 and 2 and the four auxiliary indicators calculation method, the desert comprehensive state index is calculated for every regional unit. Considering the maximum and minimum value of the index, the index is divided into three levels, as shown in Table 3.

The dominant indicator is defined as the desert comprehensive state index. The four auxiliary indicators are defined as the sand dune height, the blown sand strength, the vegetation coverage ratio and the temperature annual average difference. The division boundary of desert highway assessment is mainly drawn according to the dominant indicator and revised in fine detail by the four auxiliary indicators. By the three section name method (geographical position + affection strength + title) the Chinese deserts are classified into three regions, as shown in Table 4. The first region is named as the east medium effect region, the second as the middle medium-severe effect region and the third as the west slight-medium effect region.

**Figure 6:**
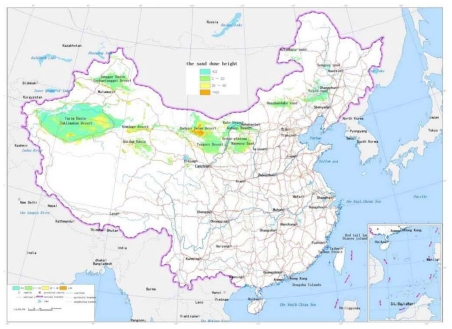
The natural region division map of desert highway assessment in China.

As shown in Figure 6, in the east medium effect region, there are continuous sand dunes. The height difference is small among all the sand dunes. During the route design, the long straight lines should be dominant, the vertical section should follow the natural geology and the low-filling embankments should be dominant. In the middle medium-severe effect region, the height differences of the sand dunes are big. The topographic features of the sand dunes are complex. The route design is limited by the longitudinal and transverse distribution plane of sand dunes. In the plane, the route trend should be parallel to the wind direction. In the longitudinal section, the route should follow the natural geology. If the route reaches the high and big sand dunes, the route should be arranged in front of the windward slope, not on the back of the lee slope.

The route must not be arranged along the top of the sand-sliding slopes. It is best for the route not to cross the lee slope of sand dunes. If this cannot be avoided, the route length should be as short as possible. In the west slight-medium effect region, there are low sand dunes and rough sand land. For the route design, the vegetation should be protected as much as possible. The low-filling embankments should be dominant for the subgrade. It is better not to excavate the land and protect the vegetation.

## 5. Project Application

The Yuling-Jingbian expressway is the first highway in desert regions of China. The length of the route is 115.98 km. The design standards are a totally closed, double direction and four-lane expressway. The designed maximum vehicle speed is 100 km/h and the width of the expressway is 26 m. The route crosses the Yulin wind-sand grass shoal area, which is defined as part of the west slight-medium effect region according to the desert assessment of the paper. The terrain is flat, sparsely populated, with good vegetation. The route design should avoid the vegetation and protect the natural environment, so the design route length is lengthened to 127 km from 115.98 km. The whole investment of the Yuling-Jingbian expressway is 2.8 billion U.S. dollars. The investment for the afforestation project and sediment control structure is nearly 0.15 billion dollars. The subgrade is mainly low-filled embankment, the maximum excavation 6.26 m and the maximum filling 2.93 m. If the embankment slope height is less than 3 meters, the slope ratio is selected as 1:3 to 1:8. If the slope height is more than 3 meters and less than 8 m, the slope ratio is selected as 1:2. The shelter-forest is constructed along both sides of the embankment, which serves as a “green corridor” for the desert highway.

## 6. Conclusions

The desert highway assessment in China is presented, based on the general desert assessment. Referring to the natural environment parameters and highway construction experience, it proposes four auxiliary indicators, which are the sand dune height, the blown sand strength, the vegetation coverage ratio and the annual average temperature difference. All the indicators are crucial to the highway construction in desert regions, which can reflect the physiognomy, climate and vegetation status. Further the dominant indicator is proposed as the desert comprehensive state index. Combining the dominant and auxiliary indicators, the index system is presented. First the natural region division is carried out according to the dominant indicator. Then the fine division boundary is revised using the four auxiliary indicators. Finally the natural region division map for desert highway assessment in China is formed. China’s deserts are classified into three regions: the east medium region, the middle medium-severe region and the west slight-medium region, which will pave the way for the highway route planning, highway design, construction, maintenance, running management and formulation of standards.

## Figures and Tables

**Table 1:** Scale value of every indicator.

Comprehensive characteristics state	Unit	Desertification intensity classification			
		Slight	Medium	Severe	Extremely severe
		1	3	6	10
Sand dune height(H)	m	<=2	2~20	20~60	>60
Vegetation coverage ratio (P)	%	>=40	40~30	30~15	<15
Blown sand strength (F)	m/(s·year)	<=2,500	2,500~3,500	3,500~4,500	>4,500
Annual average temperature difference (T)	°C	<=30	30~35	35~40	>40

**Table 2:** Contribution coefficients to the desert comprehensive state index.

	Sand dune height	Vegetation coverage ratio	Blown sand strength	Annual average temperature difference	Total
Contribution coefficient	0.3	0.3	0.28	0.12	1

**Table 3:** Effect of the desert environment on highway construction.

Desert Comprehensive state index	≤3	3~6	7~10
Level of effect on highway construction	slight	medium	severe

**Table 4:** Desert highway Natural division in China.

Name	Main Criteria and index value	Range
I the east medium effect region	The comprehensive state index is within the region [0 6] and the region [4 6] is dominant.	Hulun Beir Sandy Land Nenjiang sandy land Horqin Sandy Land Hunshandake Sand Land
II the middle medium-severe effect region	The comprehensive state index is within the region [3 10] and the region [4 6] is dominant.	Mu Us Sandy Land Kubuqi Desert Ulan Bub Sandy Land Tengger Desert Badan Jilin desert Desert of Gansu Corridor Kumtag Desert Desert of Qaidam Basin Eastern of Gurbantunggute Desert
III the west slight-medium effect region	The comprehensive state index is within the region [0 6] and the region [0 3] is dominant.	Middle and Western of Gurbantunggute Desert Taklimakan Desert
